# Motor Stereotypic Behavior Was Associated With Immune Response in Macaques: Insight From Transcriptome and Gut Microbiota Analysis

**DOI:** 10.3389/fmicb.2021.644540

**Published:** 2021-07-29

**Authors:** Xuan Pan, Fangyuan Liu, Yang Song, Hongrun Wang, Lingyun Wang, Hong Qiu, Megan Price, Jing Li

**Affiliations:** ^1^Key Laboratory of Bio-Resources and Eco-Environment (Ministry of Education), College of Life Sciences, Sichuan University, Chengdu, China; ^2^Development and Application of Human Major Disease Monkey Model Key Laboratory of Sichuan Province, Sichuan Hengshu Bio-Technology Co., Ltd., Yibin, China

**Keywords:** stereotypic behaviors, rhesus monkeys, transcriptome, gut microbiota, immune response, psychiatric disorders

## Abstract

Motor stereotypic behaviors (MSBs) are common in captive rhesus macaques (*Macaca mulatta*) and human with psychiatric diseases. However, large gaps remain in our understanding of the molecular mechanisms that mediate this behavior and whether there are similarities between human and non-human primates that exhibit this behavior, especially at gene expression and gut microbiota levels. The present study combined behavior, blood transcriptome, and gut microbiota data of two groups of captive macaques to explore this issue (i.e., MSB macaques with high MSB exhibition and those with low: control macaques). Observation data showed that MSB macaques spent the most time on MSB (33.95%), while the CONTROL macaques allocated more time to active (30.99%) and general behavior (30.0%), and only 0.97% of their time for MSB. Blood transcriptome analysis revealed 382 differentially expressed genes between the two groups, with 339 upregulated genes significantly enriched in inflammation/immune response-related pathway. We also identified upregulated pro-inflammatory genes *TNFRSF1A*, *IL1R1*, and *IL6R*. Protein–protein interaction network analysis screened nine hub genes that were all related to innate immune response, and our transcriptomic results were highly similar to findings in human psychiatric disorders. We found that there were significant differences in the beta-diversity of gut microbiota between MSB and CONTROL macaques. Of which *Phascolarctobacterium*, the producer of short chain fatty acids (SCFAs), was less abundant in MSB macaques. Meanwhile, PICRUSTs predicted that SCFAs intermediates biosynthesis and metabolic pathways were significantly downregulated in MSB macaques. Together, our study revealed that the behavioral, gene expression levels, and gut microbiota composition in MSB macaques was different to controls, and MSB was closely linked with inflammation and immune response. This work provides valuable information for future in-depth investigation of MSB and human psychiatric diseases.

## Introduction

Stereotypic behaviors, typically defined as repetitive, unvarying, and seemingly functionless behaviors (Mason, 1991), are common in millions of animals kept in captivity (Mason and Latham, 2004). Motor stereotypic behaviors (MSB) are full body repetitive behaviors and are the most commonly exhibited stereotypies. Motor stereotypies are often used as indicators of acute or chronic stress (Mason, 1991) and evaluating captive animal well-being (e.g., Novak, 2003; Gottlieb et al., 2013). Recommendations for reducing MSB have largely focused on behavioral therapy, such as positive reinforcement (Coleman and Maier, 2010), social housing (Baker et al., 2012a), environmental enrichment (Schneider et al., 1991), and visual stimulation (Platt and Novak, 1997). However, these treatments have not produced long-lasting effects or have provided minimal improvement. Consequently, there may be underlying factors that were not previously considered including complex mechanisms, such as basal ganglia dysfunction and cerebellum abnormalities in neurobiology aspect (Bechard et al., 2016; Poirier and Bateson, 2017; Wilkes and Lewis, 2018; Díez-León et al., 2019), dysregulation of neurotransmitters (Langen et al., 2011), or imbalance of intestinal flora (Zheng et al., 2016).

Rhesus macaques (*Macaca mulatta*) are extensively used in scientific research as animal models and commonly exhibit MSB (Lutz et al., 2003; Novak, 2003; Coleman and Maier, 2010; Baker et al., 2012b; Gottlieb et al., 2013, 2015). In a survey of three National Primate Research Centers, as high as 83% of singly housed rhesus macaques displayed MSB (Lutz et al., 2003; Lutz, 2018). At present, studies conducted on MSB in rhesus macaques are divided into two main areas: (1) the MSB exhibited by captive macaques, such as pacing, bouncing, rocking, swaying, and bizarre posture (Pomerantz et al., 2012; Gottlieb et al., 2013); and (2) risk factors that might be related to MSB, including time spent indoors, relocations (Gottlieb et al., 2013), blood draws, being single housed at a young age (Lutz et al., 2003), reared indoors (as opposed to outdoors), gender, age (Novak, 2003; Gottlieb et al., 2013), and other individual factors such as personality (Vandeleest et al., 2011; Gottlieb et al., 2013, 2015). While the previous work on rhesus macaques with MSB have mainly focused on the causes, types, enrichment techniques, and pathophysiology, the studies on gene expression changes in macaques exhibiting MSB are lacking, and this has limited our understanding of potential mechanisms underlying MSB. Given the wide use of non-human primates, such as rhesus macaques, in biomedical research, the study of the gene expression changes influencing MSB is of great significance for their application as model animals.

Gene expression changes are known to influence, and be influenced by, behavior (Robinson et al., 2008). For example, brain gene expression of the fish, *Betta splendens*, was changed after short-term fighting (Vu et al., 2020) and a significant number of genes were differentially expressed in rat brains after participating in social play (Alugubelly et al., 2019). Other studies have found that gene expression changes related to stereotypies including where the *Shank1* gene knockout mouse displayed repetitive behaviors (Sungur et al., 2014) and monkeys of *MECP2* transgenic overexpression exhibited a high frequency of repetitive circular locomotion and increased stress responses (Liu et al., 2016). Given the changes observed in these aforementioned genes, we speculated that MSB may lead to other changes in gene expression in rhesus macaques.

In addition, gut microbiota affects behavior *via* the microbiota-gut-brain axis (Diaz Heijtz et al., 2011). Gut microbiota is known as “the second brain” *via* a complex enteric nervous system that communicates with the brain *via* the vagus nerve (Maynard et al., 2012; Avetisyan et al., 2015; Yoo and Mazmanian, 2017). Several models of microbiota manipulation, including germ-free mice, antibiotic administration, fecal microbiota transplantation, probiotics and prebiotic, have demonstrated the important role that microbiota plays in behavior. For example, Zheng et al. (2016) transplanted “depression microbiota” derived from MDD (major depressive disorder) patients to germ-free mice and found that the mice exhibited depression-like behaviors. However, the role of gut microbiota in MSB has been little investigated.

Consequently, we aimed to investigate the relationship between MSBs, changes in gene expression and the composition of gut microbiota in captive rhesus macaques. We compared the behavior of stereotypical and normal macaques using instantaneous and focal sampling and examined the gene expression in peripheral blood cells using RNA-sequencing and gut microbiota in fecal samples using 16S rRNA sequencing. We aimed to better understand the molecular mechanisms underlying MSB and to identify potential biomarkers related to the MSB, which is essential for further research on MSB related diseases in humans and rhesus macaques.

## Materials and Methods

### Ethical Note

All observations and samplings were approved by the Sichuan University’s Animal Care Committee.

### Animals and Experimental Design

Observations and physiological sampling were conducted at Sichuan Hengshu Bio-Technology Co., Ltd. at Yibin County, Sichuan, China. Based on discussions with keepers and our own observations, five rhesus macaques displaying obvious stereotyped behaviors (i.e., MSB macaques or MSB group) and five normally behaving macaques (i.e., control macaques or control group) were selected for our behavioral assessments and molecular studies. The macaques were, on average, 4.9 years (SD = 1.1 years). During behavioral assessments (see below), subjects were randomly moved to a standard single cage (measuring 0.6 m × 0.7 m × 0.8 m) in a room with other macaques. Macaques had limited physical contact with their neighboring macaque through the metal mesh divider between cages. Although macaques did not have full physical contact and could not access other cages, they had the ability to see and groom each other. Subjects were fed three times daily with commercial monkey biscuits and daily fresh fruit or vegetables. Water was provided freely through automatic systems. None of our studied macaques were administered antibiotics during observations.

### Behavioral Data Collection and Sampling

All macaques had a 10-day habitation period to acclimate to the new conditions before we collected behavioral data. We determined behavioral classifications during this acclimation period. Behaviors were divided into six distinct behavioral categories (see Table 1 for definitions; Lutz et al., 2003; Vandeleest et al., 2011; Pomerantz et al., 2012).

**Table 1:** Ethogram.

Class	Behavior	Description
General behavior		Includes itching, grooming, feeding, drinking, and moving
Activate behavior		Includes looking around, exploration, and playing
Inactivate behavior		Includes sitting and resting
Social behavior		Includes grooming each other and chasing
Motor stereotypic behavior (MSB)	Bouncing	Repetitively using one’s hind legs or all four limbs to push oneself off the cage surface
	Flipping	Repeated forward or backwards somersaults may utilize the cage sides or ceiling
	Pacing	Repetitive walking same path in cage
	Rocking	A back and forth movement of the upper body with feet stationary
	Swaying	Holding the upper flank of the cage with one arm while swinging
Other repetitive behaviors	Hair pulling	Plucking out hair from the body using a hand
	Self-bite	Mouth-to-self contract where teeth contact the skin
	Licking cage bars	Licking the bars of the cage
	Biting cage bars	Biting the bars of the cage

We conducted behavioral observations from March 28 to May 3, 2019. To minimize disturbance of researcher and to ensure the accuracy of the data, we installed eight cameras on the wall to record the macaque behaviors. Videos were taken from 9:00 AM, when the keepers had finished cleaning the cage, and continued until 5:00 PM. Then the videos were stored in the computer for the later analysis of behavior. Videotapes were played back and recorded every 15-min. Behavioral recording work was conducted by two trained observers with scan sampling method (Altmann, 1974). Inter- and intra-rater reliability was calculated using Cohen’s Kappa for the scores awarded by the two observers. Then, data on all behavioral categories were gathered with an inter-observer reliability of greater than 90% for each discreet behavioral item. All behavioral data were recorded as frequency of occurrence.

Data were analyzed with Microsoft Excel 2016 and R software, version 3.6.3.^1^ Time budgets for each behavioral category were calculated as a proportion of observations (i.e., count of each behavior/total number of scans). We also selected a subset of data to compare the behavior changes over time. We choose 11-day data (from April 2 to April 12, *n* = 11) as the pre-acclimation phase and then 11-day data (from April 21 to May 1, *n* = 11) as the post-acclimation phase. Data distribution was tested using the Shapiro–Wilk test (*α* = 0.05). For comparison between two groups and two phases, unpaired *t*-test or Mann–Whitney *U* test was performed as appropriate. Non-parametric Kruskal-Wallis tests were used to analyze time allocation differences within groups. Data are expressed as mean ± SD. The differences were considered as statistically significant at *p* ≤ 0.05.

### RNA Extraction, Libraries Preparation, and Sequencing

We collected blood samples from the 10 macaques after the last day of behavioral data collection. The blood samples were collected using PAXgene blood RNA Tube (BD, United States) by the veterinarian, and then stored at −80°C refrigerator. Three-fold volumes of red blood cell lysate (TIANGEN, Beijing, China) were added to the blood sample and mixed every 5 min at room temperature. Peripheral blood mononuclear cells were separated from anticoagulated peripheral blood by centrifugation with Sorvall Legend X1 (Thermo Scientific, MA, United States) and then were used to extract RNA.

Total RNA was extracted by using trizol reagent (Invitrogen, Carlsbad, CA, United States), following the manufacturer’s protocol and treated with RNase-free DNase I. The extracted RNA samples were then used for the cDNA synthesis. The cDNA fragments were purified to construct the final cDNA library. The cDNA library was sequenced on the Illumina sequencing platform (Illumina HiSeq 2000) using the paired-end technology by Novogene Bioinformatics Institute, Beijing, China.

### Differential Expression Analysis and Functional Enrichment

Raw data were refined by Trim Galore software version 0.6.5^2^ to remove index, adapter, and low-quality sequences. High-quality sequences were mapped to a reference genome sequence using the Bowtie2 software version 2.4.1 (Langmead and Salzberg, 2012). The reference genome sequence and annotation file (gtf format) were downloaded from Ensembl Release 90^3^ Mmul_10. The results of mapping were sorted by samtools version 1.8 (Li et al., 2009). The counts that were equal to the number of reads mapped to each gene were counted by HTSeq version 0.12.4 (Anders et al., 2015). Due to the differences of gene length and depth of sequencing affecting counts, raw counts were normalized by DESeq2 version 1.20.0 (Love et al., 2014) of R software version 3.6.3, and the normalized counts were used to compute the expression of each gene. The impact of gender was considered, we took gender as covariate in design formula (design = ~gender + group) for differential expression analysis. Principal components analysis of sample variance indicated one outlier sample (Supplementary Figure S1), which was excluded from subsequent analyses. We used the differential expression fold change (FC) to filter differentially expressed genes (DEGs), and the filter standard was corrected value of *p* < 0.05.

Gene Ontology (GO) is an international standardized gene functional classification system, and Kyoto Encyclopedia for Genes and Genomes (KEGG) analysis can help us to understand the biological functions of genes. All DEGs were mapped to GO databases^4^ and KEGG databases^5^ by g:Profiler^6^ and KOBAS^7^ webserver, respectively (Xie et al., 2011; Reimand et al., 2016). Significance was considered as corrected *p* < 0.05.

### Protein-Protein Interaction Network Analysis

The DEGs were inputted into a protein–protein interaction (PPI) network analysis using STRING web server (STRING database v11.0, http://www.string-db.org/). We mapped the DEGs to STRING to evaluate the functional interactions among DEGs. Then, the STRING network was loaded into Cytoscape software (version 3.8.0, available at http://www.cytoscape.org/; Shannon et al., 2003) for analysis of hub genes using the plug-in cytohubba application (Chin et al., 2014). The gene with the highest number of degrees was regarded as a hub gene, which may play a critical role in the regulatory network.

### Real-Time Quantitative PCR

The expression of six DEGs were verified by quantitative real-time PCR (qPCR; including randomly selected immune related genes *IL6R*, *CD14*, *TLR6*, *TNFRSF1A*, and two other genes (*IDO1* and *IGF2R*)). The primers sequences designed with *Primer-BLAST* designing tool^8^ were shown in Supplementary Table S2. The expression level of GAPDH was used as an internal control for normalization. The qPCR program was set as: first 95°C for 30 s; followed by 95°C for 5 s and 60°C for 30 s with 40 cycles; and then 95°C for 15 s, 60°C for 60 s, and 95°C 15 s. For each sample, qPCR was performed for three technical replicates. Relative gene expression level was calculated using 2^-ΔΔCT^ method.

### 16S rRNA Sequencing of Gut Microbiota

We collected fresh fecal samples from the macaques after the last day of behavioral data collection. Collection trays were positioned under the cage and the feces were immediately transferred into sterile tubes once deposited. We then transported the fecal samples to the laboratory in dry ice and stored at −80°C refrigerator until processing. The samples were sent to Novogene Bioinformatics Institute, Beijing, China, to carry out sequencing. The V3-V4 hypervariable regions of the bacterial 16S rRNA gene was amplified with primers *338F* (5ꞌ-CCTAYGGGRBGCASCAG) and *806R* (5ꞌ-GGACTACNNGGGTATCTAAT). The prokaryotic composition of the tested samples was assessed by bioinformatic analysis of metagenomic amplicons (BioMaS) on the paired end (PE) reads generated by Illumina MiSeq sequencing.

### Sequence and Data Analysis

Quality filtering and processing of sequences was performed using the Quantitative Insights Into Microbial Ecology version 2 (QIIME2) software suite (version 2020.2; Bolyen et al., 2019) following the recommended tutorials. A feature table comprised of amplicon sequence variants (ASVs) was inferred from reads using the DADA2 algorithm (Callahan et al., 2016). Taxonomy was assigned to each representative ASVs sequence using Naïve Bayes classifiers trained against the Greengenes 13_8 99% operational taxonomic units (OTUs) reference database. Alpha diversity and beta diversity analyses were performed with q2-diversity plugin in QIIME2. Alpha diversity summarizes the microbial diversity within each sample, including Shannon index, richness index, evenness index, and Faith’s Phylogenetic Diversity (Faith-pd) index. Beta diversity measures differences between samples, including unweighted UniFrac, weighted UniFrac, Jaccard distance, and Bray–Curtis dissimilarity. In addition, we performed principal coordinate analysis (PCoA) to visualize the beta diversity of the microbiome by using custom R scripts.

To detect bacterial taxa and KEGG pathways with significantly different abundances between MSB and control samples, the linear discriminant analysis (LDA) effect size (LEfSe) analysis was used according to the online protocol (Segata et al., 2011).^9^

To explore the functional profiles of our bacterial community data set, the functional profiles of microbial communities were predicted using PICRUSt (Phylogenetic Investigation of Communities by Reconstruction of Unobserved States) according to the online protocol^10^ (Langille et al., 2013) and STAMP software packages (Minh et al., 2008). KEGG ortholog abundances were assigned and collapsed by KEGG pathway to hierarchy level 3.

## Result

### Behavior

**Figure 1:**
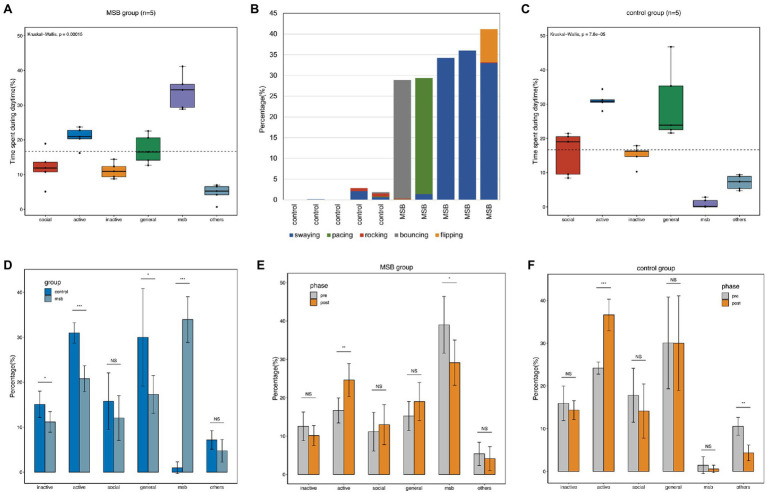
Diurnal time allocation of captive macaques. **(A)** Time budget differences between different behavioral categories in the MSB group. **(B)** The proportion of time allocated by macaques to the five different MSB types. **(C)** Time budget differences between different behavioral categories in the control group. **(D)** Time budget differences between the MSB and control groups. **(E)** Time budget differences between different phases in the MSB group. **(F)** Time budget differences between different phases in the control group. ^*^p < 0.05, ^**^p < 0.01, and ^***^p < 0.001.

We recorded 35,258 observational scans from the 10 macaques over 37 days. The most common diurnal behavior for macaques in the MSB group was MSB (33.95 ± 4.54%). The proportion of MSB was significantly higher than the other five behavioral categories (Kruskal-Wallis H = 19.03, *p* < 0.001), with MSB being more common than active behavior (20.79 ± 2.59%), general behavior (17.30 ± 3.75%), social behavior (12.05 ± 4.45%), inactive behavior (11.17 ± 2.04%), and other repetitive behaviors (4.74 ± 2.25%; Figure 1A). A total of five MSB types were observed in our study, of which swaying was the most common MSB and rocking was the least (Figure 1B). Not every macaque exhibited all five types of MSB, with individuals displaying only one to four stereotypies.

While active behavior (30.99 ± 2.04%) and general behavior (30.01 ± 9.72%) were the two most common diurnal behaviors in the control group, these macaques also exhibited MSB but only for a small proportion of their observation time (0.97 ± 1.17%). The proportion of social behavior and inactive behavior was similar, with 15.79 ± 9.72% and 15.09 ± 2.61%, respectively. The remaining 7.16 ± 1.85% was spent on the other repetitive behavior. The proportion of active or general behaviors in this group was significantly higher than other behaviors (Kruskal-Wallis H = 20.87, *p* < 0.001; Figure 1C).

The proportion of inactive (*t* = 2.36, *p* = 0.046, unpaired *t*-test), active (U(40,15) = 0, *p* < 0.001, Mann–Whitney test), and general behavior (*t* = 2.44, *p* = 0.041, unpaired *t*-test) in the control group was significantly higher than in the MSB group. Additionally, proportional time allocated to MSB was significantly lower in control group macaques (*t* = 14.06, *p* < 0.001, unpaired *t*-test; Figure 1D).

Compared with the behavior data of pre- and post-acclimation phase, the active behavior was significantly increased in both phase in MSB group (*p* = 0.011) and control group (*p* < 0.001). The proportion of MSB in MSB group was significantly decreased in the post-acclimation phase from 39.01 to 29.13% (*p* = 0.047), while other repetitive behaviors in control group was significantly decreased from 5.36 to 4.12% (*p* = 0.001; Figures 1E,F).

### Transcriptome

Blood transcriptome sequencing yielded 330 billion RNA-seq reads, with an average of 32.9 million reads per sample. After adapter cleaning, quality trimming, and duplicate- and length-filtering, 98.3% of the reads remained for analyses (Supplementary Table S1). On average, 92.9% of the quality-filtered reads were mapped to the reference genome. A total of 17,899 genes were annotated in the rhesus macaque genome (Mmul_10.98).

**Figure 2:**
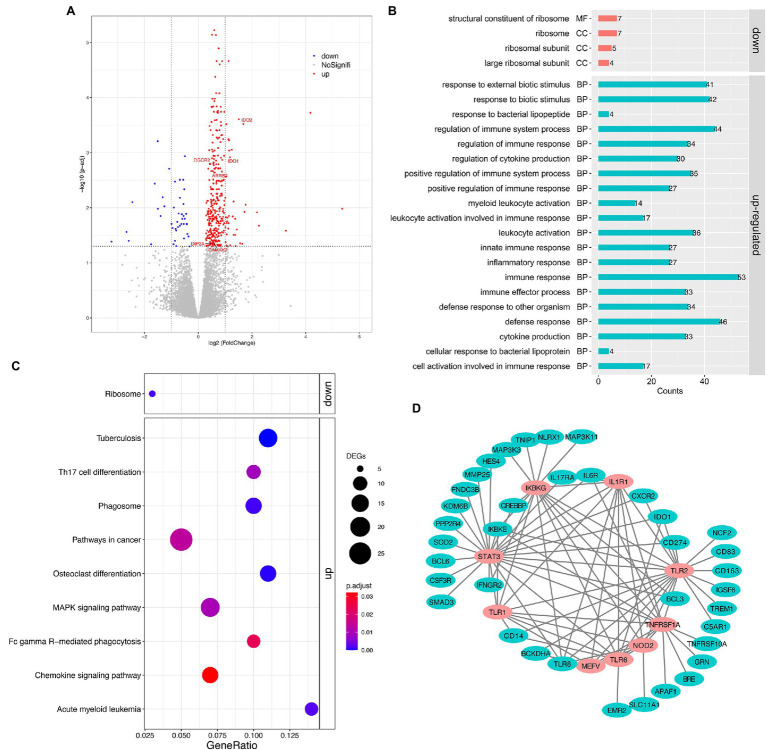
Transcriptomic analysis of the peripheral whole blood in captive macaques. **(A)** Volcano plot for expression comparisons between MSB and control individuals in peripheral blood samples. Significantly differentially upexpressed candidates (FDR < 0.05) are plotted in red, and significantly differentially downexpressed candidates (FDR < 0.05) are plotted in blue, while gray dots indicate non-significantly expressed genes. **(B)** GO enrichment analysis of DEGs. **(C)** KEGG pathway enrichment analysis of DEGs. **(D)** The sub-network of the PPI analysis of DEGs. The pink circles represent the hub genes that related to innate immune response. All the genes showed here were up-regulated genes.

Pairwise analysis of gene expression revealed a large set of significantly DEGs between the MSB and control groups, with 382 genes at a 5% false discovery rate (FDR, *q* < 0.05; Figure 2A; Supplementary Figure S2). Of these, 43 genes were downregulated and 339 genes were upregulated in the MSB group. Significantly upregulated genes included many genes related to immunity, for example, interleukin 6 receptor (*IL6R*), monocyte marker (*CD14*), perivascular macrophage marker *CD163* (*CD163*), and interleukin 8 receptor (*CXCR2*). There were several genes related to neurodevelopment, including Calcium/Calmodulin Dependent Protein Kinase Kinase 2 (*CAMKK2*), DiGeorge syndrome critical region 2 gene (*DGCR2*), and disconnected-interacting protein homolog 2 A (*DIP2A*). Genes related to neurotransmitters included Arrestin Beta2 (*ARRB2*), indoleamine 2,3-dioxygenase 1 (*IDO1*) and indoleamine 2,3-dioxygenase 2 (*IDO2*; Figure 2A). Significantly downregulated genes included: *RPS16* and *RPS26* that encode a ribosomal protein, which is a component of the 40S subunit; *RPL9* that encodes a ribosomal protein, which is a component of the 60S subunit; and *MRPL23* that encodes a mammalian mitochondrial ribosomal protein.

The upregulated DEGs in the MSB group were significantly enriched for 58 GO terms (Figure 2B; Supplementary Table S3) under the biological process’s category, including immune response (GO: 0006955, adjusted *p* < 0.001), cytokine production (GO:0001816, adjusted *p* < 0.001), inflammatory response (GO:0006954, adjust *p* < 0.001), and innate immune response (GO:0045087, adjusted *p* < 0.001). The immune system process was the most significantly enriched GO term. The downregulated DEGs in the MSB group were significantly enriched for structural constituent of ribosome (GO:0003735, adjust *p* < 0.001) by molecular function and the other three GO terms by cellular component, which all related to the ribosome.

The upregulated and downregulated genes were subjected to KEGG pathway enrichment analysis with the adjusting value of *p* cutoff of 0.05 (Figure 2C). The upregulated genes were mainly enriched in many immune-related pathways, including Chemokine signaling pathway (mcc04062, adjusted *p* = 0.03; Supplementary Figure S3A), Th17 cell differentiation (mcc04659, adjusted *p* = 0.009), Fc gamma R-mediated phagocytosis (mcc04666, adjusted *p* = 0.023), while the downregulated genes were significantly enriched in Ribosome (mcc03010, adjusted *p* = 0.001; Supplementary Figure S3B).

We obtained 364 intersections between 222 genes from the DEG PPI analysis. We then screened the hub genes of the PPI network using plus-in cytoHubba according to MCC (Maximal Clique Centrality) method, and those that scored ≥5 were selected. Subsequently, the top nine hub genes were obtained, including Innate Immunity regulator (*MEFV*), Inhibitor Of Nuclear Factor Kappa B Kinase Regulatory Subunit (*IKBKG*), Toll Like Receptor 1 (*TLR1*), Toll Like Receptor 6 (*TLR6*), Nucleotide Binding Oligomerization Domain Containing 2 (*NOD2*), TNF Receptor Superfamily Member 1A (*TNFRSF1A*), Toll Like Receptor 2 (*TLR2*), Interleukin 1 Receptor Type 1 (*IL1R1*), and Signal Transducer And Activator Of Transcription 3 (*STAT3*; Figure 2D), which were all related to immunization and participated in the innate immune response.

### The Result of Real-Time Quantitative PCR

**Figure 3:**
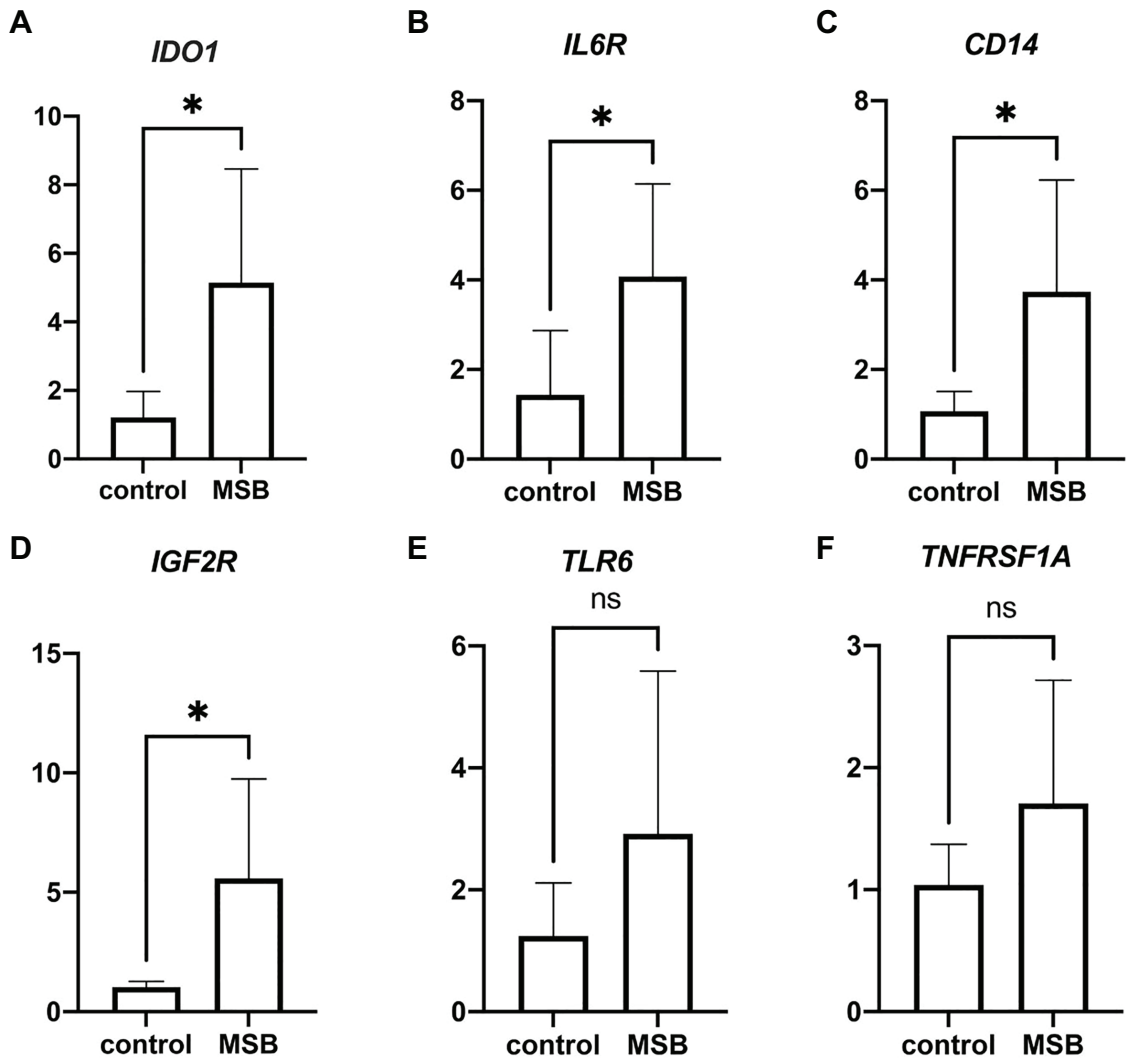
Results from quantitative real-time PCR (qPCR) validation. **(A–D)** Significant expression difference between control group and MSB group verified by qPCR of four genes. **(E,F)** Insignificant expression difference between control group and MSB group verified by qPCR of two genes. ^*^p < 0.05.

We performed real-time qPCR to verify the gene expression of blood transcriptome. We found significant increases in expression of *IDO1*, *IL6R*, *CD14*, and *IGF2R* in MSB group (Figures 3A–D), and the expression changes of these four genes were consistent with the results from blood transcriptomes. Despite there were no significant change in the expression of *TLR6* and *TNFRSF1A* (Figures 3E,F), these results showed the similar expression tendency with blood transcriptomes. The above results of qPCR indicated that the repression changes of DEGs were reliable.

### Gut Microbiota

We performed amplicon sequencing of the V3-V4 region of the 16 s rRNA gene on fecal samples from MSB and control macaques. After we filtered out the low-quality reads, a total of 1,028,775 high quality clean reads were generated from the 10 fecal samples with a mean of 102,877 ± 5,453 sequences per sample. The sequences were then identified and further grouped into 1,193 ASVs at the 99% sequence similarity level. MSB and control groups had 849 ASVs and 839 ASVs, respectively.

**Figure 4:**
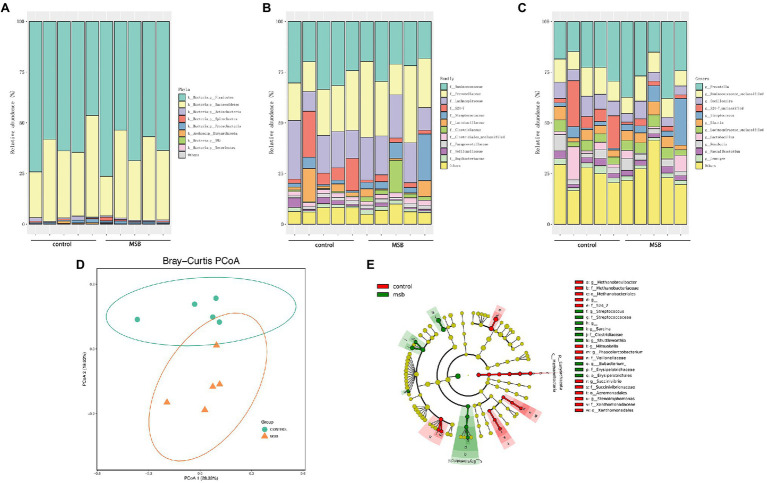
16S rRNA gene amplicons analysis. **(A)** The microbial composition between MSB and control groups at the phyla level. **(B)** The microbial composition between MSB and control groups at the family level. **(C)** The microbial composition between MSB and control groups at the genus level. **(D)** PCoA of beta-diversity using the Bray–Curtis dissimilarity metric between the MSB and control groups. Each dot represents an individual macaque. PCo1 and PCo2 represent the percentage of variance explained by each coordinate. **(E)** Cladogram of statistical differences between MSB and control group microbial populations. Red indicates increased abundance in control macaques; Green indicates increased abundance in MSB samples.

The gut microbiota of captive rhesus macaques in our study could be assigned to nine bacterial phyla. The top three dominant phyla were largely dominated by Firmicutes (46.44–76.40%), Bacteroidetes (19.47–50.11%) and Proteobacteria (0.48–1.83%), and they accounted for more than 97% of the total gut community (Figure 4A). The major components at the taxonomic family level were Ruminococcaceae (18.26–33.51%), Prevotellaceae (14.78–37.97%), and Lachnospiraceae (9.71–28.94%; Figure 4B). At the genus level, 104 genera were detected across all samples. More than 90% of these sequences belonged to 22 genera, with the dominant genera being *Prevotella* (15.12–37.97%), *Oscillospira* (4.25–10.77%), an unclassified genus from Ruminococcaceae (3.90–8.89%), and an unclassified genus from S24-7 (1.12–22.65%; Figure 4C).

In order to distinguish variations in gut microbiomes between the two groups, multiple diversity indexes were utilized. There was no significant difference in the alpha diversity between the two groups (Kruskal-Wallis test: *p* > 0.05; Supplementary Figure S4A), whereas the beta diversity exhibited significant differences between two groups. Bray-curtis distance (PERMANOVA: *p* = 0.019; Figure 4D), Jaccard (PERMANOVA: *p* = 0.012; Supplementary Figure S4B), and unweighted-Unifrac distance matrix (PERMANOVA: *p* = 0.011; Supplementary Figure S4B) indices, were statistically significant between the two groups.

**Figure 5:**
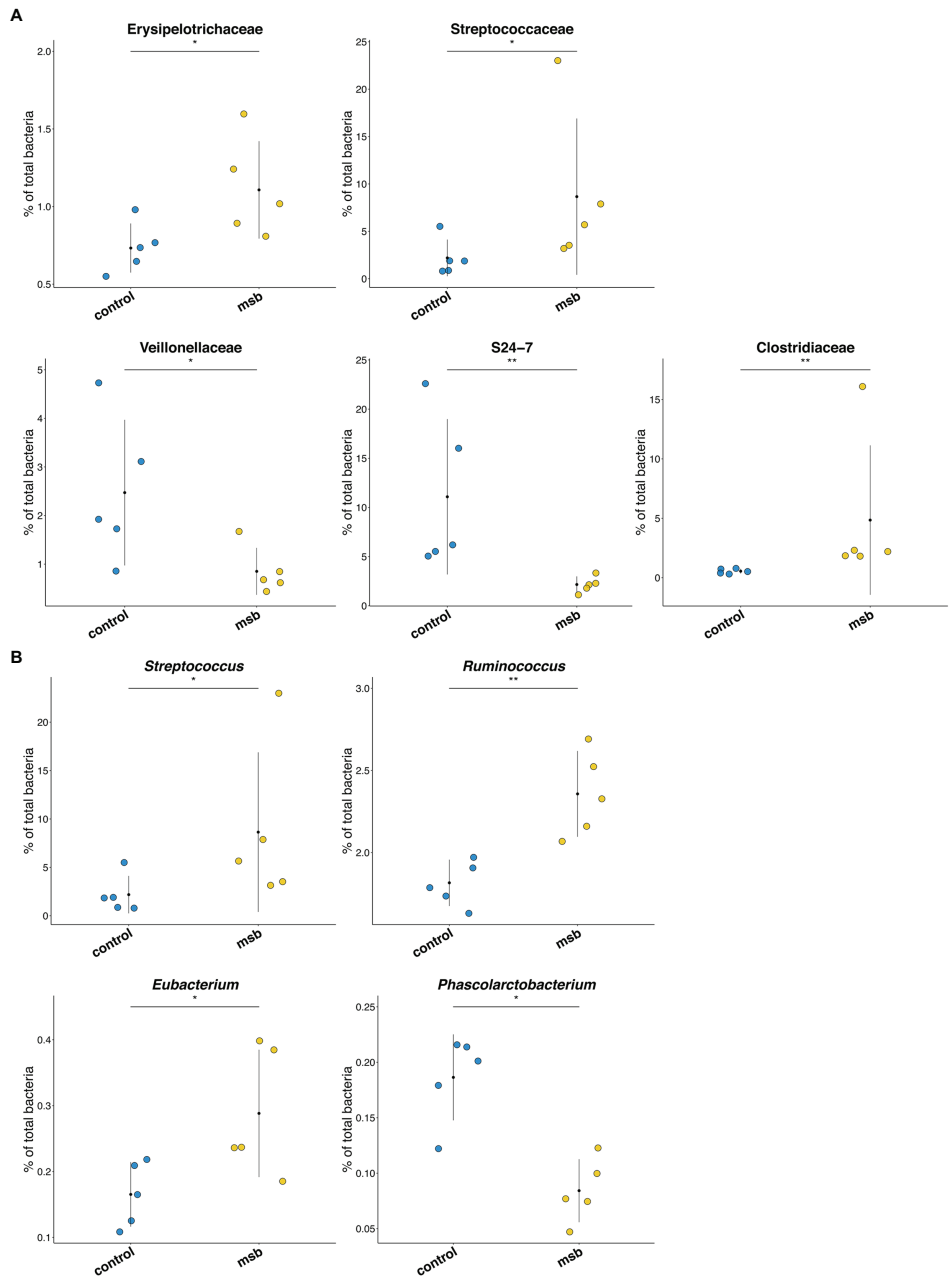
Differences in bacterial composition between the MSB and control group. **(A)** Bacterial taxa relative abundance at the family level between MSB and control groups. Horizontal points represent the mean. Error lines plotted denote SD. **(B)** Relative abundance across different genera between MSB and control groups. Horizontal points represent the mean. Error lines plotted denote SD. ^*^p < 0.05 and ^**^p < 0.01.

We performed LEfSe tests to detect differences in relative abundance of bacterial taxa across samples to explore microbial composition variation between the two groups. We focused on nine phyla and 83 genera that had an average relative abundance of 0.01% or greater across all samples (Figure 4E). At the phyla level, Euryachaeota was significantly less abundant in the MSB group. At the family level, Streptococcaceae, Clostridiaceae, and Erysipelotrichaceae were more abundant in the MSB group than the control group, while Veillonellaceae, Methanobacteriaceae, and S24-7 were less numerous in the MSB group (Figure 5A). At the genus level, MSB macaques had higher levels of ASVs in, for example, *Streptococcus*, *Ruminococcus*, and *Eubacterium*, and lower levels in genera such as *Succinivibrio*, *Stenotrophomonas*, and *Phascolarctobacterium* (Figure 5B).

**Figure 6:**
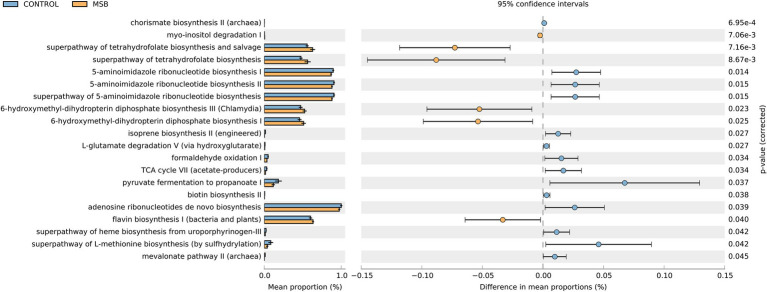
Relative abundance of the predicted metagenome genes related to KEGG pathways at level 3; orange box: MSB samples, blue box: control macaques. The terms given on the left are KEGG pathways annotated at level 3 (*p* < 0.05).

We used PICRUSt as a predictive exploratory tool to explore the different metabolic potentials between two groups. A total of 14 downregulated pathways were enriched in the MSB group, including L-methionine biosynthesis, 5-aminoimidazole ribonucleotide biosynthesis, pyruvate fermentation, and TCA cycle (acetate-producer). Meanwhile, six up-regulated pathways were enriched in the MSB group, including tetrahydrofuran biosynthesis (LDA > 2 and *p* < 0.05; Figure 6).

## Discussion

Motor stereotypic behaviors are commonly found in many captive non-human primates (Lutz et al., 2003; Pomerantz et al., 2012; Gottlieb et al., 2013; Lutz, 2018) and in human with psychiatric diseases, such as autism spectrum disorder (ASD), obsessive–compulsive disorder (OCD), and trichotillomania (Lewis and Kim, 2009; Langen et al., 2011; Lutz, 2014; Zabegalov et al., 2019). However, to date, very little is known about the molecular mechanisms underlying these disorder behaviors or whether there are similarities between human and non-human primates that exhibit stereotypic behaviors, in particular gene expression and gut microbiota variation. The present study combined behavioral, transcriptome, and gut microbiota data from MSB macaques to investigate the underlying mechanism of MSB and the relatedness to human psychiatric diseases to provide new insights in an animal model that is the focus of extensive psychiatric disease research.

### Expression Changes in the Nervous System Related Genes in MSB Macaques

Given the profound behavior difference between individuals of the MSB and control groups, changes in gene expression may have played an important role in observed behavioral differences. Comparison of peripheral blood transcriptome data revealed that there were 382 DEGs between MSB and control groups, of which 339 were upregulated and 43 were downregulated DEGs in the MSB group.

Several upregulated genes in the MSB group were related to neurodevelopment, such as *CAMKK2*, *DGCR2*, and *DIP2A*, which might contribute to MSB in rhesus macaques. *CAMKK2*, belonging to the serine/threonine protein kinase family, is involved in the CAM pathway (Anderson et al., 1998). *CAMKK2* gene has the highest expression in brain and plays key roles in brain function, including neurodevelopment (e.g., neurite outgrowth; Wayman et al., 2004; Cao et al., 2011), neuronal differentiation and migration (Cao et al., 2011) and synapse formation, and cognitive function such as learning and memory (Mizuno et al., 2007). Altered *CAMKK2* activity has been found to be strongly associated with many behavioral disorders (Sayad et al., 2018). Meanwhile, there is evidence that *CAMKK2* is a susceptibility gene of Schizophrenia (Luo et al., 2014). *DGCR2* encodes an activity-dependent adhesion protein (Kajiwara et al., 1996) and participates in neurodevelopment (Maynard et al., 2003). Evidence indicated that abnormal behavior and locomotor activity were significantly reduced in *DGCR2* knockout mice in open field tests (Mugikura et al., 2016). Additionally, *DGCR2* is associated with Schizophrenia (Shifman et al., 2006; Xu et al., 2011). *DIP2A* is known to be involved in acetylated coenzyme A (AcCoA) synthesis and is primarily expressed in the brain regions with abundant pyramidal neurons (Mukhopadhyay et al., 2002; Zhang et al., 2015; Ma et al., 2019a). *DIP2A* knockout mice exhibited autism-like behaviors, including excessive repetitive behaviors and defects in social novelty (Ma et al., 2019a). In addition, *DIPA2* was suggested as a candidate gene for autism (Egger et al., 2014; Wang et al., 2016; Ma et al., 2019b).

Meanwhile, MSB macaques had more highly expressed genes related to neurotransmitters than the control group including *ARRB2*, *IDO1*, and *IDO2*. *ARRB2* encodes β-arrestin 2 and is an adaptor protein that is important for receptor regulation belonging to both dopaminergic and opioid systems (Schmid and Bohn, 2009; Skinbjerg et al., 2009). *ARRB2* levels is a potential biomarker for depression and Alzheimer’s disease (Avissar et al., 2004; Golan et al., 2013; Mendez-David et al., 2013; Thathiah et al., 2013; Petit et al., 2018). *IDO1* and *IDO2*, which encode the enzyme indoleamine 2,3-dioxygenase (IDO), were overexpressed in MSB group samples. IDO activity is mainly induced by the proinflammatory cytokine IFN-γ and converts tryptophan into kynurenine and during overstimulation may lead to lowered tryptophan concentrations (Taylor and Feng, 1991; MacKenzie et al., 1999; Fallarino et al., 2003), resulting in reduced 5-HT content in the brain (Mellor and Munn, 2004; Wichers et al., 2005; Miura et al., 2008). These neurotransmitters, including dopamine, 5-HT, and GABA are related to repetitive behavior (Schoenecker and Heller, 2001, 2003; Kjaer et al., 2004). Neurotransmitters can directly or indirectly affect the activity of the basal nerve node, thus inducing repetitive behavior (Langen et al., 2011). In addition, these neurotransmitters have been linked with many mood disorders and contribute to the development of stress-related mood disorders (Schiepers et al., 2005; Krishnadas and Cavanagh, 2012; Bortolato et al., 2015), which are accompanied by the performance of anxiety-like behavior or depressive-like behavior. Further studies are necessary to validate the role of these genes in MSB, such as gene overexpression and gene knock-out.

### Inflammation and Immune Response in MSB Macaques

We found that upregulated DEGs in the MSB group compared to the control group were significantly enriched in immune-related pathways and GO terms (e.g., Chemokine signaling pathway, Th17 cell differentiation, inflammation response, and innate immune response). Meanwhile, we identified nine hub genes from the DEGs that were associated with immunization, in particular, to the innate immune response. Genes encoding important pro-inflammatory cytokines IL-1β, IL-6, and TNF were expressed significantly higher in MSB macaques than in controls indicating the severity of inflammation in the MSB macaques. Collectively, the results indicated that the immune response, especially innate immunity, was activated in MSB macaques. This activation may be a manifestation of stress, since MSB was absent in wild animals, being captive was an isolation stressful experience for the MSB individuals (Mason, 1991). Stress significantly affects immune function, with chronic and acute stressors being associated with peripheral immune activation and inducing the release of IL-1β, IL-6, TNF-α, and other pro-inflammatory cytokines (Menard et al., 2017; Mileva et al., 2017; Niraula et al., 2018; Maltz et al., 2019; Tsyglakova et al., 2019). The release of pro-inflammatory cytokines during inflammatory processes can disrupt blood–brain barrier and infiltrate the CNS (Huffman et al., 2018) and can also affect the brain *via* neural (mainly vagal) pathways, interacting with cytokine receptors on cerebral endothelial cells and/or microglial activation (D’Mello and Swain, 2017). Subsequently, the interaction disrupts brain circuits, which can control responses such as motivation, motor activity, anxiety, excitement, and vigilance (Bergink et al., 2014; Delpech et al., 2016; Johnson and Kaffman, 2018; Reus et al., 2018), and consequently evoke changes in behavior (Saunderson et al., 2016), including anxiety- or depressive-like behavior (e.g., stereotypic behavior). In addition, in the same captive environment, some individuals exhibit obvious MSB, while others not. This may be related to individual variation (Koolhaas et al., 2010), since the regulation of behavior is influenced by both genes and environment interactions (Ledón-Rettig et al., 2013). However, more experiments are required to investigate this difference.

Activation of the immune response, especially inflammation, has been implicated in the pathophysiology of several psychiatric disorders in humans (e.g., Schizophrenia, ASD, Depression, Bipolar disorder, OCD, and Anxiety disorder; Hinze-Selch and Pollmacher, 2001; Wilson et al., 2002; Kaplin et al., 2005; Kerr et al., 2005; Leonard and Myint, 2006; Marazziti and Dell’Osso, 2015; Dell’Osso et al., 2016). Several inflammation cytokines, such as TNF and IL-6 in serum, have been considered as sensitive biomarkers in identifying a predisposition for psychiatric disorder or as a diagnosis of a psychiatric disorder (Pape et al., 2019). Similar to humans with psychiatric disorders, our MSB macaques showed a high level of immune responses and over-expression of inflammatory cytokines. This may indicate a similar molecular mechanism between MSB macaques and humans suffering from psychiatric disorders. Consequently, our results suggest that MSB macaques are a suitable animal model to study and evaluate human psychiatric disorders.

### Gut Microbiota in MSB Macaques

Gut microbiota influence brain and behavioral functions *via* the microbiota-gut-brain axis (Diaz Heijtz et al., 2011), in turn, the brain influences microbiota composition in the gut through effects on digestive and immune molecules (Foster and McVey Neufeld, 2013), thereby becoming a bidirectional interaction. We compared gut microbiota variation between the MSB group and control group to investigate the relatedness of gut microbiota and MSB in macaques. We found a significant difference in beta diversity between the two groups, suggesting that the gut microbiota composition was significantly different in MSB macaques relative to control group, implying an imbalance of gut microbiota of the MSB macaques. There is substantial evidence that the alteration of gut microbiota can trigger inflammation (Scher et al., 2013; Boulangé et al., 2016; Strati et al., 2016). For instance, bacteria from the Erysipelotrichaceae family was reported to be correlated to stomach and intestinal inflammation in human (Burrello et al., 2019; Chen et al., 2020). Overall, the alteration of gut microbiota in MSB macaques may induce inflammation, while this result and the transcriptome results corroborate each other to some extent.

We also identified that *Phascolarctobacterium* was less abundant in the MSB group, which is an organism that produces short chain fatty acid (SCFA) of propionate (Zhang et al., 2018). PICRUSTs analysis showed the important intermediates of SCFAs (pyruvate and acetate) were downregulated in the MSB group. SCFAs are the most abundant microbial metabolites in the intestine and play crucial roles in microbiota-gut-brain communication (Dalile et al., 2019), regulating important signaling molecules of mental disease (De Vadder et al., 2014; Li et al., 2018; Tian et al., 2019) and the anti-inflammatory response in the central nervous system (Cavaglieri et al., 2003; Fung et al., 2017). Animal experiments have shown that SCFA supplements reduced anxiety-like behavior in the open field test and depressive-like behavior in the forced swim test (van de Wouw et al., 2018). Our results suggested that the abundance of SCFA producing bacteria, such as *Phascolarctobacterium*, might affect inflammatory response thus be closely related to behavioral disorder in MSB macaques.

In addition, the changes of gut microbiota in MSB macaques showed similarity to that of human with psychiatric disease. The relative abundances of bacteria from families Streptococcaceaey, Erysipelotrichaceae, and Clostridiaceae and from genera *Streptococcus*, *Ruminococcus*, and *Eubacterium* were higher, and the abundances of Veillonellaceae families were lower in MSB group. The abundance of these bacteria has also been reported to be different in MDD patients (Jiang et al., 2015; Zheng et al., 2016; Lin et al., 2017; Chen et al., 2018; Barandouzi et al., 2020). This may also reflect a similar molecular mechanism from gut microbiota aspect between MSB macaques and people with mental illness.

## Conclusion

In conclusion, the present study combined data from behavior, blood transcriptome, and gut microbiota to investigate the underlying mechanism of MSB and the relationship of the behavior to gene expression and gut microbiota in rhesus macaques. Five types of stereotypic behavior were recorded and MSB macaques spent about 34% of their diurnal time on MSB. Comparison of blood transcriptome from MSB and control groups revealed DEGs related to neurodevelopment and neurotransmitters. Importantly, it was demonstrated that immune response, especially innate immunity, was over-activated in MSB macaques. Genes of pro-inflammatory cytokines IL-1β, IL-6, and TNF were significantly upregulated in MSB macaques, which was similar to results from human psychiatric disorder studies. Moreover, we found that gut microbiota composition was significantly different in MSB macaques and the metabolic pathway of SCFAs intermediates was significantly downregulated. The present study demonstrated that MSB was associated with inflammation/immune response, and this result was similar with humans suffering from psychiatric disorders in gene expression and gut microbiota variation. Further investigations based on MSB macaques will greatly benefit research on repetitive stereotyped behavior in humans with psychiatric disorders.

## Data Availability Statement

The data presented in the study are deposited in NCBI repository, accession number: PRJNA670320.

## Ethics Statement

The animal study was reviewed and approved by the Sichuan University’s Animal Care Committee.

## Author Contributions

JL conceived and designed the experiments. XP collected the data, conducted the primary analyses, and wrote the manuscript. FL helped with the validation experiment. YS, HW, LW, and HQ helped with data collection and analyses. XP, MP, and JL reviewed and corrected the manuscript. All authors contributed to the article and approved the submitted version.

## Conflict of Interest

HW and LW were employed by the company Sichuan Hengshu Bio-Technology Co., Ltd.

The remaining authors declare that the research was conducted in the absence of any commercial or financial relationships that could be construed as a potential conflict of interest.

## Publisher’s Note

All claims expressed in this article are solely those of the authors and do not necessarily represent those of their affiliated organizations, or those of the publisher, the editors and the reviewers. Any product that may be evaluated in this article, or claim that may be made by its manufacturer, is not guaranteed or endorsed by the publisher.

## Footnotes

^1^ http://www.R-project.org

^2^ http://www.bioinformatics.babraham.ac.uk/projects/trim_galore/

^3^ http://asia.ensembl.org/index.html

^4^ http://www.geneontology.org/

^5^ http://www.genome.jp/kegg/

^6^ https://biit.cs.ut.ee/gprofiler/gost

^7^ http://kobas.cbi.pku.edu.cn/

^8^ https://www.ncbi.nlm.nih.gov/tools/primer-blast/

^9^ https://huttenhower.sph.harvard.edu/galaxy/

^10^ http://picrust.github.io/picrust/
