# Supplementary material for: Motor Stereotypic Behavior Was Associated With Immune Response in Macaques: Insight From Transcriptome and Gut Microbiota Analysis
Source: Front Microbiol. 2021 Jul 29;12:644540. doi: 10.3389/fmicb.2021.644540 (PMC8360393; doi:10.3389/fmicb.2021.644540)
Supplement: Supplementary file 1 [file Data_Sheet_1.docx]

**Supplementary Table 1. The mapping results of different samples**

| **group** | **Sample Name** | **Sex** | **GC%** | **Q20(%)** | **Q30(%)** | **Raw Reads** | **Clean Reads** | **overall alignment rate (%)** |
| --- | --- | --- | --- | --- | --- | --- | --- | --- |
| **control** | HS112 | female | 54.3 | 97.78 | 94.03 | 32,300,406 | 32,015,544 | 93.97 |
|  | HS111 | female | 53.63 | 97.7 | 93.92 | 38,275,426 | 37,611,964 | 92.95 |
|  | HS205 | female | 53.92 | 97.5 | 93.46 | 30,968,519 | 30,071,064 | 92.03 |
|  | HS204 | female | 53.26 | 97.83 | 94.09 | 38,527,510 | 38,138,185 | 93.12 |
|  | HS402 | female | 53.8 | 97.88 | 94.23 | 39,604,247 | 38,955,400 | 92.98 |
| **MSB** | HS102 | female | 53.87 | 97.86 | 94.19 | 32,379,390 | 32,110,755 | 92.87 |
|  | HS104 | female | 55.52 | 98 | 94.56 | 27,858,006 | 27,457,717 | 91.46 |
|  | HS203 | female | 55.12 | 98.09 | 94.69 | 29,715,958 | 28,955,482 | 94.52 |
|  | HS406 | male | 54.22 | 97.94 | 94.31 | 30,504,651 | 30,119,086 | 93.72 |
|  | HS212 | female | 52.78 | 97.61 | 93.56 | 29,620,863 | 28,915,944 | 92 |

**Supplementary Table 2. The primer information of qRT-PCR**

| **Gene** | **Primer (5' to 3')** |
| --- | --- |
| IDO1 | TGCACGATCACGTAAACCCA |
|  | ATAGCTGGGGGTTGCCTTTC |
| CD14 | AGAACCTTGTGAGCTGGACG |
|  | GATCGACGCGCGTTAGAAAC |
| IL6R | TCACTGTGTCATCCACGACG |
|  | TGGATTCTGTCCAAGGCGTG |
| TLR6 | AGCTTCCATTTTGTTTGCCTT |
|  | CTTTGGTTTTCGGCGGTAGG |
| TNFRSF1A | TGCAAGATCCCCTTCCAACC |
|  | GAGGGTAGCACCAAGTAGGC |
| GAPDH | AGCCGCATTTTCTCTTGCATC |
|  | GACCAAATCCGTTGACTCCG |
| IGF2R | CCAGGTGGCAAAAAGTGACG |
|  | CGTTGTTATAAGGCGTGCCG |

**Supplementary Table 3. The result of GO enrichment analysis**

| **comparison** | source | term_name | term_id | p.adjust | term_size | count |
| --- | --- | --- | --- | --- | --- | --- |
| **MSB vs control up-regulated** | GO:BP | immune response | GO:0006955 | 0.000 | 1024 | 53 |
|  | GO:BP | immune effector process | GO:0002252 | 0.000 | 498 | 33 |
|  | GO:BP | leukocyte activation | GO:0045321 | 0.000 | 612 | 36 |
|  | GO:BP | regulation of immune response | GO:0050776 | 0.000 | 568 | 34 |
|  | GO:BP | defense response | GO:0006952 | 0.000 | 979 | 46 |
|  | GO:BP | cytokine production | GO:0001816 | 0.000 | 582 | 33 |
|  | GO:BP | defense response to other organism | GO:0098542 | 0.000 | 624 | 34 |
|  | GO:BP | response to biotic stimulus | GO:0009607 | 0.000 | 883 | 42 |
|  | GO:BP | response to external biotic stimulus | GO:0043207 | 0.000 | 858 | 41 |
|  | GO:BP | leukocyte activation involved in immune response | GO:0002366 | 0.000 | 175 | 17 |
|  | GO:BP | cell activation involved in immune response | GO:0002263 | 0.000 | 178 | 17 |
|  | GO:BP | inflammatory response | GO:0006954 | 0.000 | 435 | 27 |
|  | GO:BP | regulation of cytokine production | GO:0001817 | 0.000 | 523 | 30 |
|  | GO:BP | innate immune response | GO:0045087 | 0.000 | 441 | 27 |
|  | GO:BP | regulation of immune system process | GO:0002682 | 0.000 | 995 | 44 |
|  | GO:BP | positive regulation of immune response | GO:0050778 | 0.000 | 460 | 27 |
|  | GO:BP | positive regulation of immune system process | GO:0002684 | 0.000 | 712 | 35 |
|  | GO:BP | myeloid leukocyte activation | GO:0002274 | 0.001 | 138 | 14 |
|  | GO:BP | cellular response to bacterial lipoprotein | GO:0071220 | 0.001 | 5 | 4 |
|  | GO:BP | response to bacterial lipopeptide | GO:0070339 | 0.001 | 5 | 4 |
|  | GO:BP | cellular response to bacterial lipopeptide | GO:0071221 | 0.001 | 5 | 4 |
|  | GO:BP | positive regulation of cytokine production | GO:0001819 | 0.001 | 344 | 22 |
|  | GO:BP | T cell activation involved in immune response | GO:0002286 | 0.002 | 72 | 10 |
|  | GO:BP | CD4-positive, alpha-beta T cell activation | GO:0035710 | 0.002 | 74 | 10 |
|  | GO:BP | response to bacterial lipoprotein | GO:0032493 | 0.003 | 6 | 4 |
|  | GO:BP | response to cytokine | GO:0034097 | 0.005 | 626 | 30 |
|  | GO:BP | lymphocyte activation | GO:0046649 | 0.009 | 514 | 26 |
|  | GO:BP | lymphocyte activation involved in immune response | GO:0002285 | 0.010 | 128 | 12 |
|  | GO:BP | detection of bacterial lipopeptide | GO:0070340 | 0.012 | 3 | 3 |
|  | GO:BP | cellular response to triacyl bacterial lipopeptide | GO:0071727 | 0.012 | 3 | 3 |
|  | GO:BP | response to triacyl bacterial lipopeptide | GO:0071725 | 0.012 | 3 | 3 |
|  | GO:BP | MyD88-dependent toll-like receptor signaling pathway | GO:0002755 | 0.016 | 16 | 5 |
|  | GO:BP | pattern recognition receptor signaling pathway | GO:0002221 | 0.020 | 115 | 11 |
|  | GO:BP | alpha-beta T cell activation | GO:0046631 | 0.022 | 116 | 11 |
|  | GO:BP | innate immune response-activating signal transduction | GO:0002758 | 0.022 | 116 | 11 |
|  | GO:BP | leukocyte mediated immunity | GO:0002443 | 0.023 | 239 | 16 |
|  | GO:BP | regulation of defense response | GO:0031347 | 0.023 | 445 | 23 |
|  | GO:BP | immune response-activating signal transduction | GO:0002757 | 0.025 | 268 | 17 |
|  | GO:BP | response to interferon-gamma | GO:0034341 | 0.026 | 97 | 10 |
|  | GO:BP | detection of molecule of bacterial origin | GO:0032490 | 0.027 | 9 | 4 |
|  | GO:BP | regulation of innate immune response | GO:0045088 | 0.028 | 216 | 15 |
|  | GO:BP | vacuolar acidification | GO:0007035 | 0.030 | 18 | 5 |
|  | GO:BP | positive regulation of cell death | GO:0010942 | 0.034 | 425 | 22 |
|  | GO:BP | type 2 immune response | GO:0042092 | 0.039 | 31 | 6 |
|  | GO:BP | response to molecule of bacterial origin | GO:0002237 | 0.041 | 197 | 14 |
|  | GO:BP | positive regulation of apoptotic process | GO:0043065 | 0.043 | 400 | 21 |
|  | GO:BP | immune response-regulating signaling pathway | GO:0002764 | 0.045 | 281 | 17 |
|  | GO:BP | negative regulation of cytokine production | GO:0001818 | 0.046 | 199 | 14 |
|  | GO:BP | T-helper cell differentiation | GO:0042093 | 0.048 | 47 | 7 |
|  | GO:BP | detection of bacterial lipoprotein | GO:0042494 | 0.048 | 4 | 3 |
|  | GO:BP | cellular response to diacyl bacterial lipopeptide | GO:0071726 | 0.048 | 4 | 3 |
|  | GO:BP | response to diacyl bacterial lipopeptide | GO:0071724 | 0.048 | 4 | 3 |
|  | GO:BP | positive regulation of programmed cell death | GO:0043068 | 0.049 | 403 | 21 |
| **MSB vs control down-regulated** | GO:MF | structural constituent of ribosome | GO:0003735 | 0.001 | 271 | 7 |
|  | GO:CC | ribosome | GO:0005840 | 0.001 | 326 | 7 |
|  | GO:CC | ribosomal subunit | GO:0044391 | 0.019 | 214 | 5 |
|  | GO:CC | large ribosomal subunit | GO:0015934 | 0.037 | 131 | 4 |

**Supplementary Figure 1 Principal components analysis of RNA-seq samples.** Count data for all the variant genes across all samples was subjected to principal components analysis. **A)** PC2 versus PC1. The single sample (HS204) is an outlier with respect to all other samples in PC1. **B)** PC2 versus PC3. Samples cluster somewhat by treatment group; control (red) and MSB (blue).


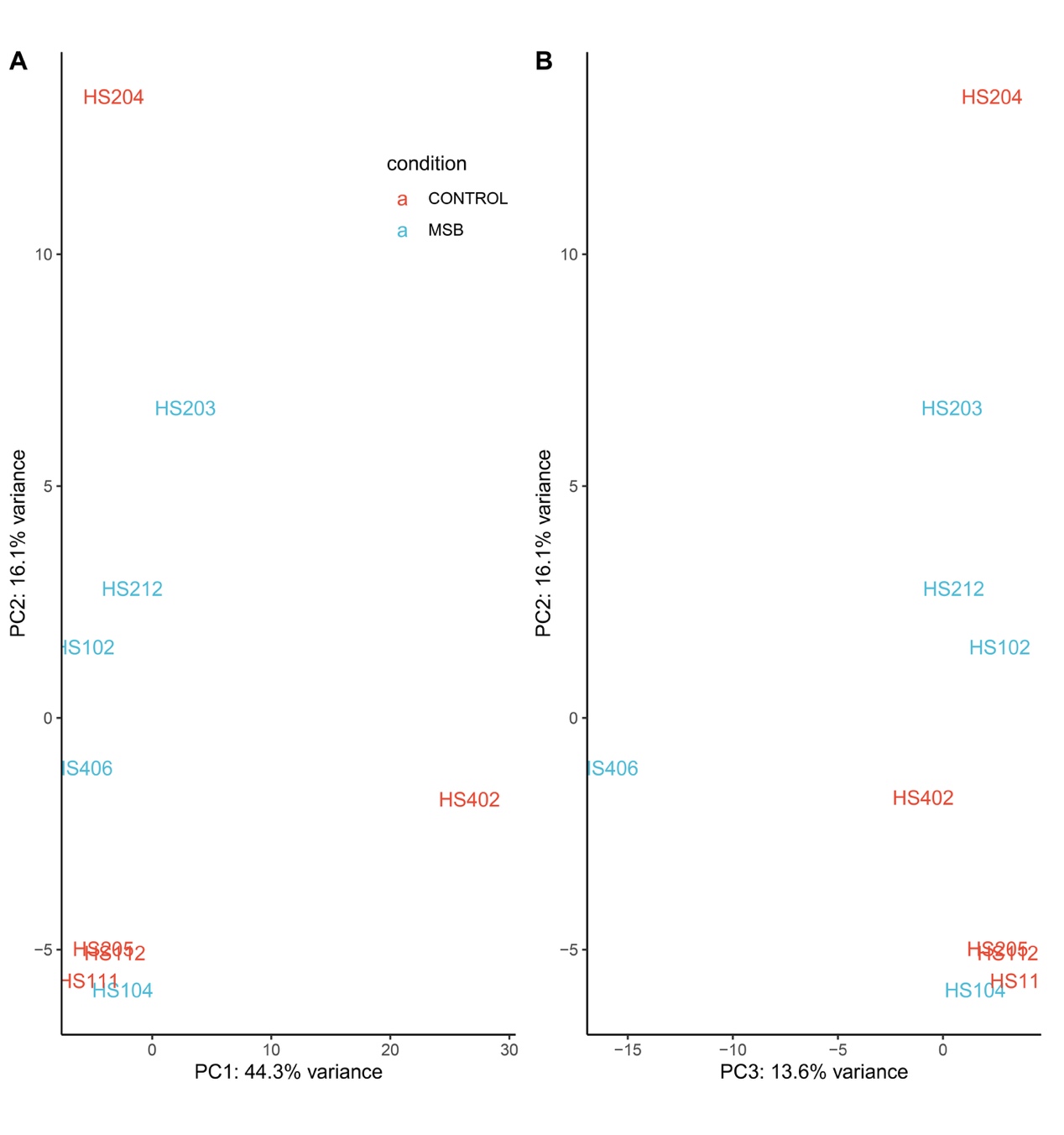


**Supplementary Figure 2 Heat map plot of DEGs using TPM expression value of genes by adopting hierarchical clustering method.** Each column represents a specimen and each row represents a gene. Red color indicates genes that were up-regulated and blue color indicates genes that were down-regulated.

**
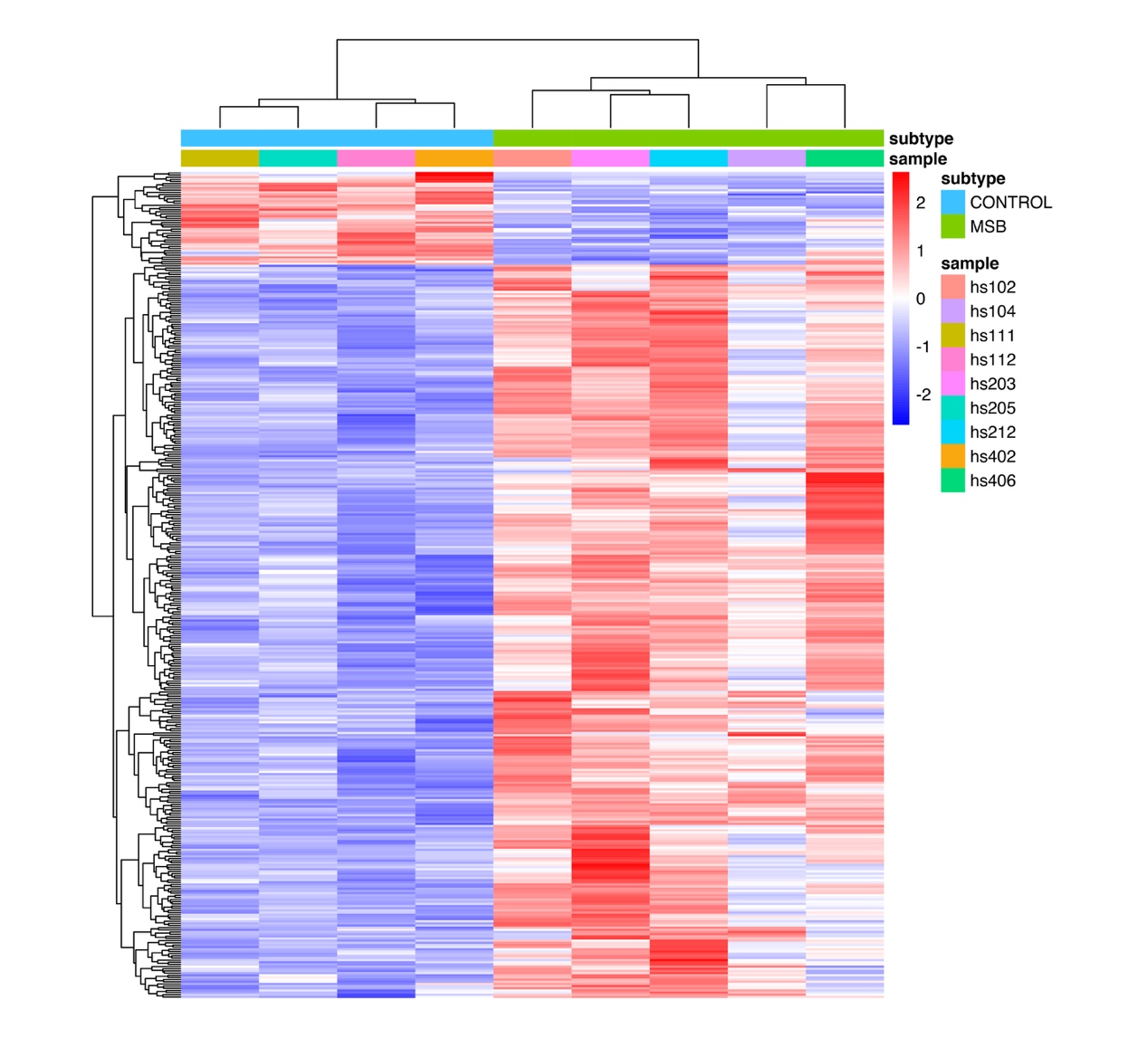
**

**Supplementary Figure 3 Differentially expressed genes between MSB and control group in pathways.** **(A)** Ribosome pathway. Significantly differentially expressed genes are in red font. **(B)** Chemokine signaling pathway. Significantly differentially expressed genes are in red font.


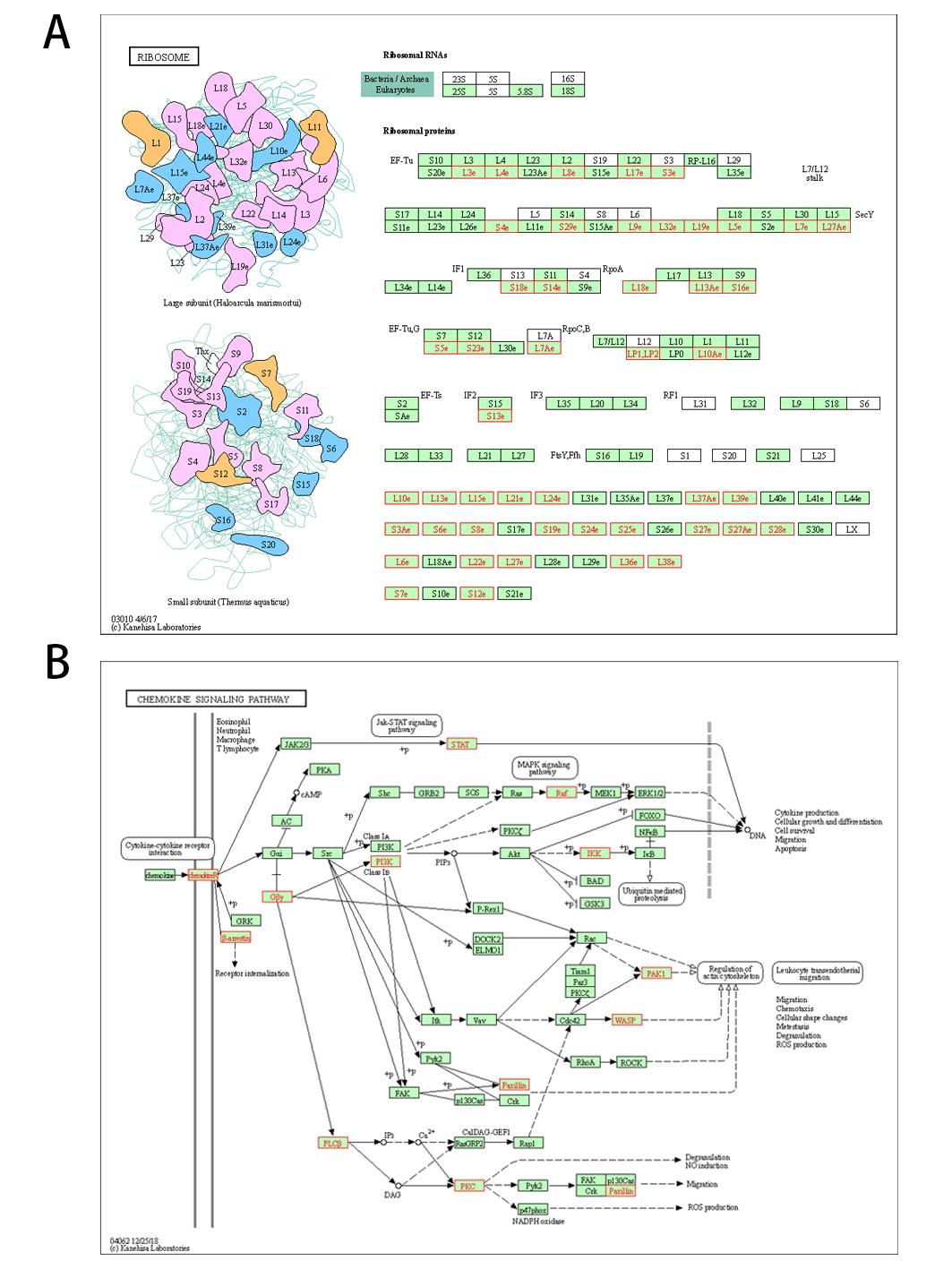


**Supplementary Figure 4 Alpha and Beta diversity. (A)** Variations in alpha diversity between MSB and control. Comparison of 16S in Shannon index, OUT amount, faith_pd index, evenness index. **(B)** PCoA plot based on Jaccard distances and unweighted UniFrac distances

**
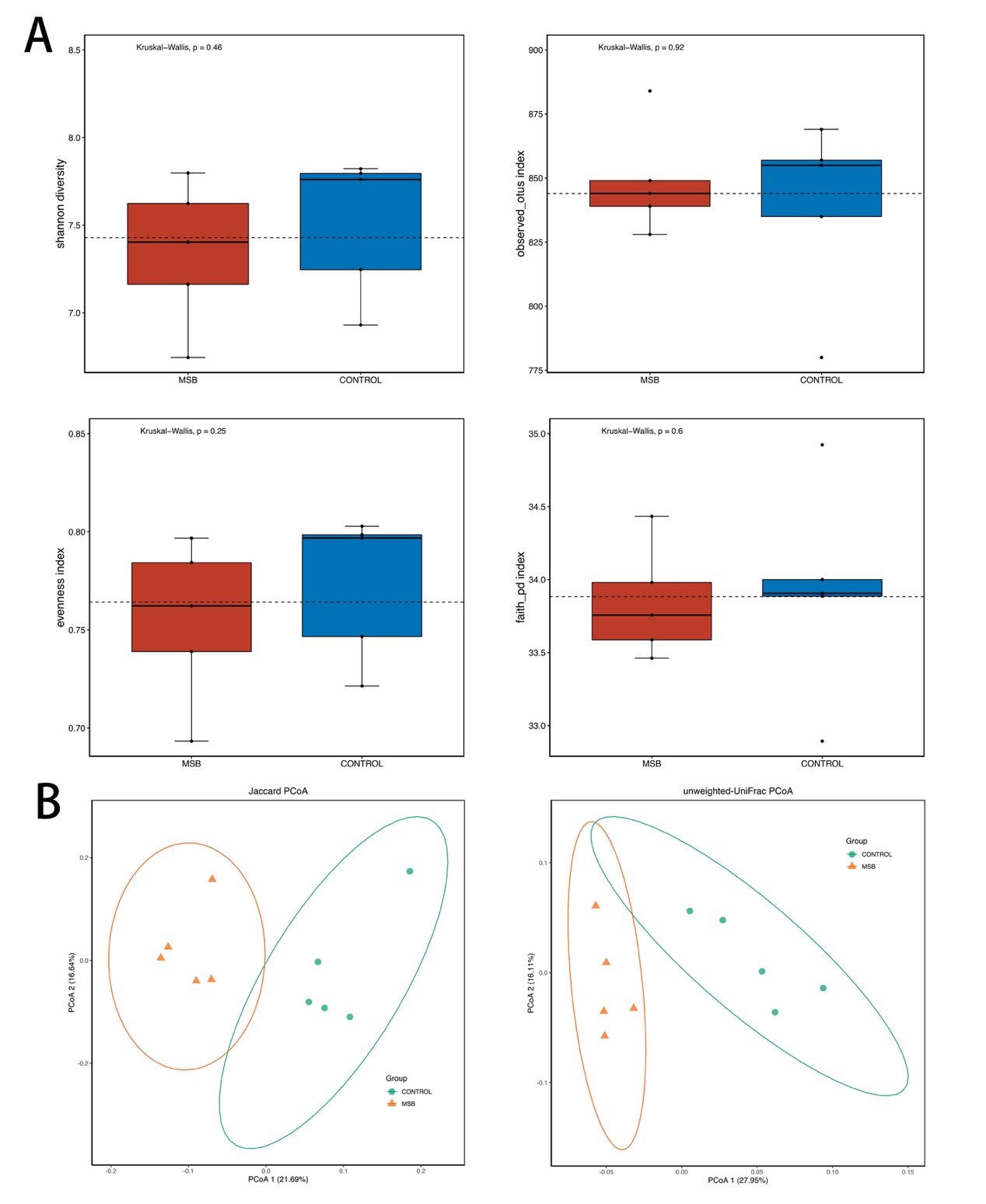
**
